# Mitochondrial dysfunctions in T cells: focus on inflammatory bowel disease

**DOI:** 10.3389/fimmu.2023.1219422

**Published:** 2023-09-22

**Authors:** Hoyul Lee, Jae-Han Jeon, Eun Soo Kim

**Affiliations:** ^1^ Research Institute of Aging and Metabolism, Kyungpook National University, Daegu, Republic of Korea; ^2^ Department of Internal Medicine, School of Medicine, Kyungpook National University, Kyungpook National University Chilgok Hospital, Daegu, Republic of Korea; ^3^ Division of Gastroenterology, Department of Internal Medicine, Kyungpook National University, Kyungpook National University Hospital, Daegu, Republic of Korea

**Keywords:** mitochondria, IBD - inflammatory bowel disease, immunometabolism, T cell, treatment, inflammation

## Abstract

Mitochondria has emerged as a critical ruler of metabolic reprogramming in immune responses and inflammation. In the context of colitogenic T cells and IBD, there has been increasing research interest in the metabolic pathways of glycolysis, pyruvate oxidation, and glutaminolysis. These pathways have been shown to play a crucial role in the metabolic reprogramming of colitogenic T cells, leading to increased inflammatory cytokine production and tissue damage. In addition to metabolic reprogramming, mitochondrial dysfunction has also been implicated in the pathogenesis of IBD. Studies have shown that colitogenic T cells exhibit impaired mitochondrial respiration, elevated levels of mROS, alterations in calcium homeostasis, impaired mitochondrial biogenesis, and aberrant mitochondria-associated membrane formation. Here, we discuss our current knowledge of the metabolic reprogramming and mitochondrial dysfunctions in colitogenic T cells, as well as the potential therapeutic applications for treating IBD with evidence from animal experiments.

## 1 Introduction

Inflammatory bowel disease (IBD) is a multifactorial immune disorder, characterized by chronic relapsing inflammation of the gastrointestinal tract accompanied with impaired immune homeostasis resulting from inappropriate and persistent activation of the mucosal immune system. Crohn’s disease (CD) and ulcerative colitis (UC) are the two major forms of the condition. Despite the accumulating evidences that immune dysregulation, intestinal microbiota, environmental factors, and genetic susceptibility are implicated in the pathogenesis of IBD (1), the exact causes remain unclear. The intestinal tract is the largest immune organ in the body, composing of complex immune-cell populations with a persistent exposure to the luminal antigens and pathogens. Therefore, immune homeostasis is essential for tolerance to luminal antigens and protection against pathogens.

**Figure 1:**
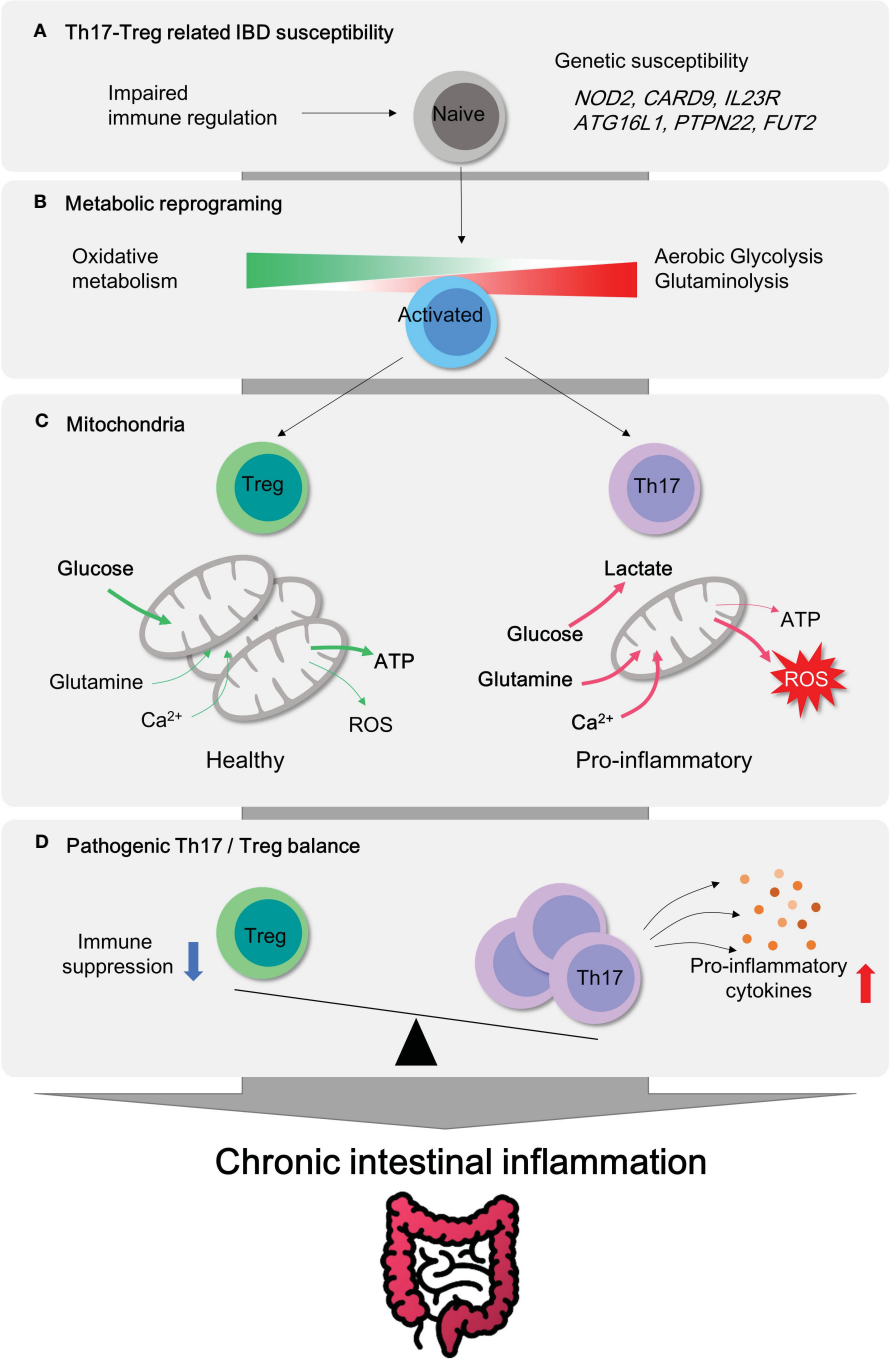
Metabolic Reprogramming and mitochondrial activity in CD4^+^ T subsets for pathogenesis of IBD. **(A)** Genetic susceptibility factors can trigger T cell-mediated immune dysregulation, leading to the onset of intestinal inflammation. **(B)** Pro-inflammatory CD4^+^ T cells become hyper-activated and demand metabolic reprogramming to meet their cellular needs for proliferation and effector functions. This metabolic shift involves the utilization of aerobic glycolysis, glutaminolysis, and mitochondrial oxidative respiration. **(C)** Regulatory T cells possess a higher number of healthy mitochondria that can efficiently utilize glucose oxidation to produce ATP without excess ROS generation. In contrast, pro-inflammatory Th17 cells rely on aerobic glycolysis and glutamine to fuel mitochondria and produce lactate. **(D)** Treg cells play a critical role in maintaining immune homeostasis in the gut mucosa by inhibiting immune responses. The imbalance between Treg and Th17 cells is a significant factor observed in patients with autoimmune diseases such as inflammatory bowel disease. Thus, targeting this imbalance could be an important strategy for the treatment of IBD.

The aberrant mucosal infiltration by innate and adaptive immune cells has been considered as a key player in the pathogenesis of IBD. According to the GWAS (genome-wide association study) based on human studies, *NOD2* (pattern recognition receptor), *CARD9* (inflammation), *IL23R* (Th17 cell responses), *ATG16L1* (autophagy), *PTPN22* (T cell activation) and *FUT2* (microbiome) are well known causative IBD genes (2, 3). This set of identified susceptibility genes for IBD simply implies vulnerability or hyper-activation of innate and adaptive immune systems (**Figure 1A**), although the opening inflammatory response is thought to remove foreign luminal antigens.

**Figure 2:**
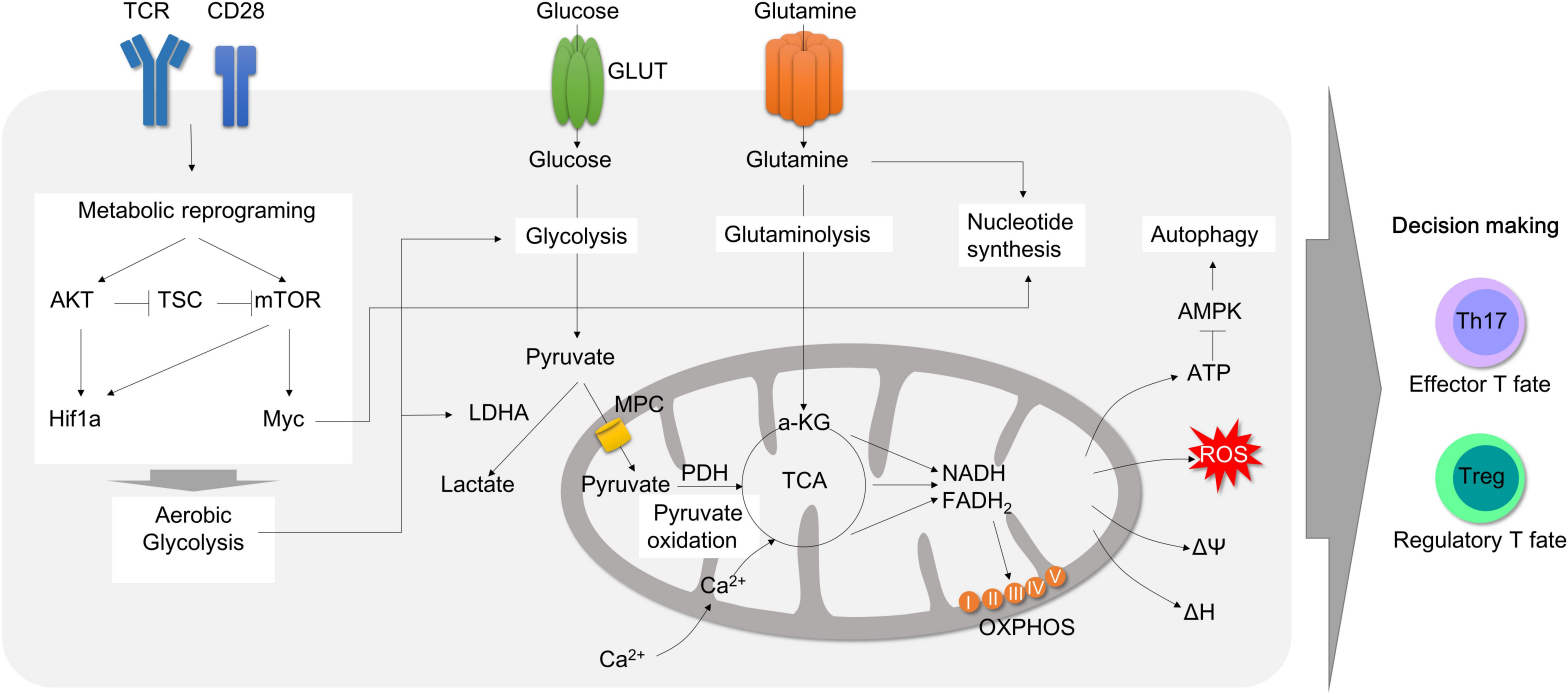
Mitochondrial involvement in T cell activation and metabolic reprogramming during inflammation. T cell activation by T cell receptor with CD28 promotes metabolic reprogramming. CD28 activation leads to AKT and mTOR activation. Consequently, Hif1α and Myc transcription factors are upregulated, which accelerates aerobic glycolysis by increasing glucose uptake and lactate secretion to rapidly generate cellular ATP and carbon building blocks. Upon activation, pyruvate oxidation is decreased and glutaminolysis rather fuels TCA cycles in mitochondria in order to produce ATP. Glutamine is also rapidly consumed to process *de novo* nucleotide synthesis. Metabolic adaptation during T cell activation consequently leads to mitochondrial instability followed by mitochondrial ROS production, which can promote effector T cell polarization. Therefore, mitochondria is a pivot to all T cell metabolic reprogramming upon activation and differentiation. TCR, T cell receptor; GLUT, glucose transporter; AKT, protein kinase B; mTOR, mammalian target of rapamycin; TSC, tuberous sclerosis complex; LDHA, lactate dehydrogenase A; MPC, mitochondria pyruvate carrier; TCA, tricarboxylic acid cycle; OXPHOS, oxidative phosphorylation; AMPK, AMP-activated protein kinase.

Uncontrolled activation of effector CD4^+^ T cell responses (Th1, Th2 and Th17) and defects in immunosuppressive activity by regulatory T cell (Treg) in lamina propria (LP) were well characterized in IBD, including both CD (4) and UC (5). In line with this, recent studies using single cell RNA sequencing have revealed that distinct subsets of CD4^+^ T cells are considered to be an essential contributing factor in the immune landscape of IBD despite heterogeneity of T cells (6–8). Depletion of CD4^+^ T by chimeric monoclonal anti-CD4 antibody is affirmative in treating patients with Crohn’s disease (9). The ablation of tissue resident memory CD4^+^ T cells protects from experimental colitis in mice (10). Transcriptomic analysis reveals that human intestinal CD4^+^ T cells display exclusive gene signatures from circulating CD4^+^ T cells, such as differential chemokine and activation gene, Th17-related transcription factors, tumor necrosis factor (TNF) receptor signaling pathways (11). These clinical observations advocate a noteworthy role of CD4^+^ T cells in IBD. Moreover, colitogenic CD4^+^ T cells have been shown to undergo metabolic changes that support their pathogenicity. For instance, hypoxia-inducible factor 1 alpha (Hif1α) transcription is exclusively over-expressed in lamina propria CD4^+^ T cells, as compared to the intestinal epithelial CD4^+^ T and circulating CD4^+^ T cells (11). As gut tissue resident memory CD8^+^ T cells exhibit distinct metabolic signatures to the naive T cells (12), colitogenic tissue resident memory CD4^+^ T cells (10) are perhaps expected to experience similar metabolic rewiring (**Figure 1**, **2**).

The last two decade, there have been numerous research papers published in terms of immunometabolism. T cells face extensive metabolic changes to sustain the energy generation to achieve activation, clonal expansion, differentiation, effector cytokine production, and survival in inflammatory environments. Naïve and resting T cells utilize mitochondrial oxidative respiration coupled to TCA (tricarboxylic acid) cycle, OXPHOS (oxidative phosphorylation) and adenosine triphosphate (ATP) biosynthesis for a stable energy generation using a limited nutrient source. Fatty acid oxidation is especially prominent in naïve, memory and regulatory T cells.

Once engaged with antigen recognition or T cell receptor (TCR) activation by CD3 and co-stimulatory receptor CD28, T cell rapidly changes in their metabolic program toward glycolysis. Although glycolysis is not so efficient for ATP production, only 2 ATP molecules per glucose molecule, compared to mitochondrial respiration, which produces 36 ATP molecules per glucose molecule, glycolysis can rapidly substitute the cellular building blocks for proliferation, differentiation and effector function. This metabolic change is known as ‘aerobic glycolysis’ or the ‘*Warburg effects’*, which was first described phenomenon in cancer pathogenesis. Later, mitochondrial dysfunction appears to be a prominent cause of aerobic glycolysis. Today, *Warburg effects* and mitochondrial dysfunction gained attention again and furthermore extended to immunology field (**Figures 1B, C**).

Mitochondria have a central role in energy metabolism and cellular homeostasis. Primarily, mitochondria provide the bioenergetics ATP as a power plant and regulate metabolic activity within the cell. Upon activation, T cells expand mitochondrial mass along with the mitochondrial ribosomal proteins, OXPHOS proteins, and one-carbon metabolism (13, 14). Besides, mitochondria contribute to cellular calcium homeostasis, apoptosis regulation, reactive oxygen species (ROS) generation, and inflammatory signaling pathways. Given the accumulated understanding of mitochondrial alterations in the process of T cell activation and Th17/Treg differentiation (**Figure 1D**), it is still a puzzle whether manipulating mitochondrial function and metabolism could modulate the inflammatory signaling of these cells and alleviate the symptoms of IBD. Recently, several reviews have been published describing how immune cell metabolism is associated with inflammatory responses in autoimmunity (15–19) and the role of mitochondria in intestinal epithelial defense regarding pathogenesis of IBD (20–22). In this review, we examine recent studies that have implicated aerobic glycolysis and mitochondrial dysfunctions in the pathogenesis of IBD (**Figure 1**). Our analysis focuses on CD4^+^ T cells, which have been identified as a key factor in the disease’s progression. We have provided experimental evidence that specifically highlights these findings (**Tables 1**–**3**).

**Table 1:** Targeting CD4^+^ T cell glucose metabolism for treating IBD - evidence from an *in vivo* animal study.

Target mechanism	Treatment	Colitis animal model	Effects or MoA^!^	Colitis outcome^#^	References
Aerobic glycolysis	High-glucose diet	DSS-induced colitis Adoptive T cell transfer colitis	Glucose availability mROS production Th17 differentiation	Deterioration	(23, 24)
	*Glut1*-OE Treg	Adoptive T cell transfer colitis	Diminished Treg function	Deterioration	(25)
	Ritonavir	NOD-scid IL-2Rγnull colitis	Glut transporter inhibition	Amelioration	(26)
	*Glut1*-depleted CD4	Piroxicam-induced adoptive T cell transfer colitis	Aerobic glycolysis in CD4 T cells	Amelioration	(27)
	*Glut3*-depleted CD4	Adoptive T cell transfer colitis	Aerobic glycolysis Availability of Acetyl-CoA and citrate Th17 differentiation	Amelioration	(28)
	*Hk2*-deficient CD4	IL-10 KO spontaneous colitis	Aerobic glycolysis	Amelioration	(29)^+^
mTOR	TSC1 deleted CD4	Adoptive T cell transfer colitis	mTOR activation Th1 and Th17 differentiation Reduced Treg function	Deterioration	(30)
	Rapamycin	Adoptive T cell transfer colitis DSS-induced colitis TNBS-induced colitis	mTOR inhibition	Amelioration	(31–36)
	Arctigenin	DSS-induced colitis	mTORC1 inhibition	Amelioration	(37)
AMPK	Metformin	DSS-induced colitis	AMPK activation	Amelioration	(38)
Hif1α	*Hif1a*-deficient T cell	DSS-induced colitis	Reduced Treg function Th17 differentiation	Deterioration	(39)
	*Vhl*-deficient Treg	Adoptive T cell transfer colitis	Hif1α stabilization Th1 differentiation	Deterioration	(40)
	PX-478	DNBS-induced colitis	Hif1α inhibition Impaired epithelial regeneration	Deterioration	(41)
	DMOG	DSS-induced colitis	Hif1α activation Enhanced barrier function	Amelioration	(42, 43)
	FG-4497	TNBS-induced colitis	Hif1α activation Enhanced barrier function	Amelioration	(44)
	CG-598	DSS-induced colitis	Hif1α activation Enhanced barrier function	Amelioration	(45)
Myc	*Myc* deleted Treg	Spontaneous colitis	Abnormal neonatal Treg development	Deterioration	(46)

^!^MOA stands for Mode Of Action.

^#^Disease severity or disease progression in the treated group is ‘ameliorated’ or ‘deteriorated’ as compared to the control group.

^+^The role of HK2 in T cell viability, activation, proliferation, differentiation, aerobic glycolysis, and mitochondrial respiration is dispensable in vitro. Nevertheless, CD4-specific deletion of HK2 reduces colitis in spontaneous colitis model of IL-10 KO mice.

**Table 2:** Targeting CD4^+^ T pyruvate and glutamine metabolism for treating IBD - evidence from an *in vivo* animal study.

Target mechanism	Treatment	Colitis animal model	Effects or MoA^!^	Colitis outcome^#^	References
Pyruvate oxidation	Ethyl pyruvate	TNBS-induced colitis	Pyruvate availability	Amelioration	(47)
	DCA	Adoptive T cell transfer colitis	PDK inhibition and PDH activation Treg differentiation Th17 reduction	Amelioration ^^^	(48)
	*Pdk4*-deficient T cell	DSS-induced colitis Adoptive T cell transfer colitis	PDK inhibition and PDH activation Reduced aerobic glycolysis	Amelioration	(49)
	GM-10395	DSS-induced colitis	PDK inhibition and PDH activation Reduced aerobic glycolysis	Amelioration	(49)
Glutaminolysis	Glutamine-supplemented diet	DSS-induced colitis	Glutamine availability	Amelioration	(50)
	Glutamine-depleted iTreg	Adoptive T cell transfer colitis	Glutamine depreviation iTreg differentiation Th1 and Th17 reduction	Amelioration	(51)
	*Gls*-deficient CD4	Adoptive T cell transfer colitis	Glutaminolysis inhibition Treg differentiation Th17 reduction Exhausted Th1 Histone modification	Amelioration	(52)
	BPTES	IL-10 KO spontaneous colitis	Glutaminolysis inhibition Th17 reduction Treg differentiation	Amelioration	(53)

^!^MOA stands for Mode Of Action.

^#^Disease severity or disease progression in the treated group is ‘ameliorated’ or ‘deteriorated’ as compared to the control group.

^^^Although disease severity was not affected in this model, the infiltration of proinflammatory T cells was significantly reduced in the gut.

**Table 3:** Targeting CD4^+^ T mitochondrial fitness for treating IBD - evidence from an *in vivo* animal study.

Target mechanism	Treatment	Colitis animal model	Effects or MoA^!^	Colitis outcome^#^	References
Mitochondrial biogenesis	*Tfam*-deficient CD4	DSS-induced colitis	Impaired mitochondrial biogenesis Polarization to Th1-like cells	Deterioration	(14)
	*Tfam*-deficient Treg	Adoptive T cell transfer colitis	Impaired mitochondrial biogenesis Reduced Treg function	Deterioration	(54)
	*Il15* deficient Rag mice	T cell transfer colitis	Impaired mitochondrial biogenesis Reduced Treg function	Deterioration	(55)
	*Il15ra* deficient Treg	T cell transfer colitis	Impaired mitochondrial biogenesis Reduced Treg function	Deterioration	(55)
Mitochondrial calcium	Ruthehium Red Ru360	TNBS-induced colitis	MCU inhibition	Amelioration	(56)
	*Pdk4*-deficient T cell	DSS-induced colitis and T cell transfer colitis	MAM inhibition SOCE decrease Reduction in calcium signaling	Amelioration	(49)
	GM-10395	DSS-induced colitis	MAM inhibition SOCE decrease Reduction in calcium signaling	Amelioration	(49)
	VBIT-4, VBIT-12	DSS-induced colitis and TNBS-induced colitis	VDAC inhibition	Amelioration	(57–59)
OXPHOS	Oligomycin	TNBS-induced colitis	ATP synthase inhibition Reduced Th17 differentiation	Amelioration	(60)

^!^MOA stands for Mode Of Action.

^#^Disease severity or disease progression in the treated group is ‘ameliorated’ or ‘deteriorated’ as compared to the control group.

## 2 Targeting T cell metabolism for treating colitis

### 2.1 Aerobic glycolysis in colitogenic T cells

CD3/CD28 co-stimulation in a naïve CD4^+^ T cells during TCR activation induces glycolysis (**Figure 2**) while producing lactate by increasing the expression of glucose transporter (61), hexokinase 2 (HK2) (29), and consumption of glucose (61), and prevents it from becoming anergy. Inhibition of glycolysis by pan-hexokinase blocker, 2-dehydroxy-D-glucose (2-DG), during T cell activation impedes cytokine secretion co-related with mTOR signaling pathway (62). Moreover, glucose availability is a key factor for effector T cell functions. Glucose availability significantly affects secretion of IL-2 and IFN-γ (63, 64), aerobic glycolysis (65), Ca^2+^/NFAT signaling pathway (64), proliferation, and viability (66) in T cells. Therefore, targeting elevated glycolysis in T cells could be fascinating approaches in treating inflammatory diseases like rheumatoid arthritis, multiple sclerosis (MS) and systemic lupus erythematosus (67).

#### 2.1.1 Glucose availability

In patients with UC, there is a higher prevalence of hyperglycemia than in controls (68). Interestingly, patients who have both psoriasis and IBD have a significantly higher prevalence rate of diabetes than those who have sole psoriasis (69). Moreover, patients with IBD and diabetes mellitus exhibit a significantly higher levels of C-reactive protein, erythrocyte sedimentation rate, eosinophil counts, monocyte counts in blood (70). Furthermore, use of metformin, a medication for the treatment of type 2 diabetes, known as an 5’ adenosine monophosphate-activated protein kinase (AMPK) activator, significantly reduces the hazardous ratio of new-onset IBD by almost a half (71), and positively prevents dextran sulfate sodium (DSS)-induced colitis in mice (38). These clinical observations may suggest that systemic glucose metabolism and intolerance is notably associated with IBD.

Hyper-glycolysis in cells often leads to mitochondrial dysfunctions by a hyper-polarizing the mitochondrial membrane potential, and oversupply of mitochondrial ROS (mROS). A high glucose concentration induces more mROS, and drives Th17 cell generation. Accordingly, the treatment of the ROS scavenger, N-acetyl-L-cysteine (NAC), or the mitochondria-targeted anti-oxidant, mitoquinone (MitoQ), in T cells significantly inhibits Th17 cell differentiation even in the presence of high glucose (23).

**Figure 3:**
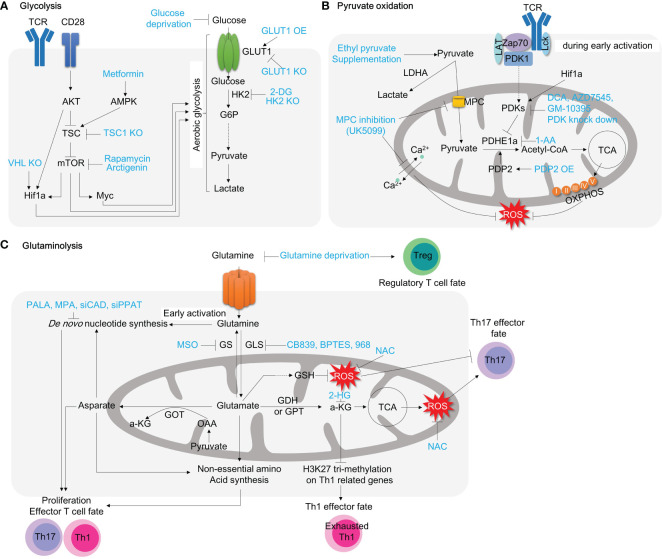
Targeting T cell metabolic reprogramming for inflammation treatment. **(A)** Targeting general aerobic glycolysis drivers, such as mTOR, Myc, and Hif1a, with inhibitors like rapamycin, arctigenin, and 10058-F4, has demonstrated anti-inflammatory effects in autoimmune CD4^+^ T cells. Inhibiting the glucose transporter GLUT1 on the plasma membrane also restricts glycolysis, which slows T cell activation. Additionally, inhibiting HK2, the first glycolytic enzyme converting glucose to G6P, with 2-DG significantly decreases T cell activation. **(B)** Pyruvate oxidation can be enhanced by pyruvate supplementation. Inhibiting the MPC with compounds like UK5099 not only induces aerobic glycolysis by increasing pyruvate levels in the cytosol but also lowers mitochondrial calcium concentration, resulting in significant mitochondrial dysfunction in CD4^+^ T cells. Inhibiting PDHE1α with 1-AA alters mitochondrial pyruvate oxidation and can impact T cell development. Conversely, promoting pyruvate oxidation by inhibiting PDK with compounds like DCA, AZD7545, and GM10395 may be a potential therapeutic strategy for intestinal inflammation. Glut3 depletion reduces acetyl-CoA availability and histone acetylation **(C)** Glutaminolysis is associated with mitochondrial function and T cell activation. Glutamine serves as a source for *de novo* nucleotide synthesis during T cell activation and effector T cell polarization. Inhibiting *de novo* nucleotide synthesis with compounds like PALA and MPA or withdrawing glutamine with compounds like MSO or glutamine deprivation suppresses effector T cell polarization. Glutaminase inhibition with compounds like CB839, BPTES, and 968 leads to alterations in Th1/Th17 polarization and shows promise as a highly effective approach for inflammation treatment. TCR, T cell receptor; AKT, protein kinase B; TSC, tuberous sclerosis complex; mTOR, mammalian target of rapamycin; Hif1a, hypoxia-inducible factor 1; HK2, hexokinase 2; G6P, glucose-6-phosphate; GLUT1, glucose transporter 1; 2-DG, 2-dehydroxy-D-glucose; LDHA, lactate dehydrogenase A; MPC, mitochondria pyruvate carrier; PDK, pyruvate dehydrogenase kinase; LAT, Linker for activation of T cells; TCA, tricarboxylic acid cycle; PDP2, pyruvate dehydrogenase phosphatase 2; DCA, dichloroacetate; GS, glutamine synthetase;GLS, glutaminase; GSH, glutathione; PALA, N-(phosphonacetyl)-L-aspartate; MPA, mycophenolic acid; MSO, methionine sulfoximine; NAC, N-acetylcysteine; 2-HG, 2-Hydroxyglutarate; a-KG, alpha ketoglutarate; GDH, glutamate dehydrogenase; GPT, glutamate pyruvate transaminase; GOT, glutamate OAA transaminase; OAA, oxaloacetate.

In animal models of colitis, high glucose availability has been shown to exacerbate the development of inflammation. Colonic expression of glycolysis-associated proteins such as HK2, lactate dehydrogenase A (LDHA), phosphate fructose kinase, and c-MYC are evidently enriched in inflamed tissues of DSS-induced colitis mouse model (72). Likewise, a high-sugar diet clearly deteriorates lymphocyte infiltration, epithelial damage, and cytokine expressions in the gut after colitic DSS challenges in mice (24). High glucose consumption (10% in a drinking water) also exacerbates T cell transfer colitis model in mice by increasing proinflammatory Th17 populations in the gut (23). Conversely, overexpressing a transgenic glucose transporter (Glut) 1 receptor in regulatory T cells fails to achieve disease remission in an adoptive T cell transfer colitis model (25) (**Figure 3A**; **Table 1**).

Glucose restriction achieves an effective prevention of colitis disease. Treatment of potential Glut blocker, Ritonavir, has been reported to ameliorate the disease severity in NOD-*scid* IL-2Rγ^null^ colitis animal model (26). Glut1-depleted CD4^+^ T cells fail to trigger intestinal inflammation in nonsteroidal anti-inflammatory drug-induced T cell transfer colitis model in mice (27). Adoptive transfer of Glut3-deficient T cells fail to induce intestinal inflammation (28). T cell specific-HK2 deficiency partially recovers intestinal inflammation in a spontaneous colitis model of IL-10 knockout (KO) mice, although HK2-deficient CD4^+^ T cells appear to have normal proliferation, viability, activation and differentiation *in vitro* (29) (**Figure 3A**; **Table 1**).

#### 2.1.2 Transcriptional control of glucose metabolism

This metabolic shifts from OXPHOS to glycolysis reliance often accompanies induction of the mammalian target of rapamycin (mTOR), c-Myc, and Hif1α pathways (73). The importance of these factors in regulating T cell metabolism and effector functions is well described in the previous literature and review (73, 74).

Tuberous sclerosis 1 (TSC1) is a negative regulator of mTORC1. Constitutive activation of mTORC1 by CD4^+^ specific deletion of TSC1 promotes Th1 and Th17 cell differentiation and suppresses immunosuppressive activity of Treg, resulting in an enhanced disease severity in an adoptive T cell transfer colitis model (30). Conversely, mTOR deficient T cells fail to differentiate into effector T cells, including Th1, Th2, and Th17 (75). The therapeutic efficacy of rapamycin, a classic pharmacological inhibitor of mTORC1, has been substantially demonstrated to ameliorate intestinal inflammations in experimental animal colitis models in a diverse perspective (31–36). Another pharmacological mTORC1 inhibitor, arctigenin, has been reported to decrease Th1 and Th17 cell differentiation, leading to amelioration of disease severity in DSS-induced colitis model (37) (**Figure 3A**).

The activation of mTOR signaling pathway further stimulates the activity of Hif1α, a master regulator of oxygen homeostasis. Although Hif1α-deficient T cells have better cellular growth and proliferation in response to TCR engagement with IL-3 (76), HIF1α–mediated metabolic reprogramming toward ‘aerobic glycolysis’ (76, 77) promotes Th17 cell differentiation and declines Treg cell differentiation in both *in vitro* experiment, and *in vivo* animal model of MS (77, 78) (**Figure 3A**). In patients with CD, and UC, Hif1α expression is highly augmented in the inflamed colonic mucosa (39), especially in Th17 cells (79). These experimental evidences may suggest that HIF-signaling pathway is hypothetically to be a fascinating therapeutic target. Despite this, the modulation of HIF levels for IBD has several limitations to induce clinical responses. First, the role of Hif1α in regulatory T cells seems to be debatable in an animal model of colitis (39, 40, 80, 81). That is presumably because Hif1α under hypoxic condition not only facilitates glycolysis but also paradoxically protects mitochondria from ROS by reducing complex I activity, pyruvate oxidation, autophagy, and mtDNA encoded mRNA levels (82). Certainly, T cell specific deletion of Hif1α significantly downregulates Foxp3 over IL-17 expression, leading to the uncontrolled immune responses in DSS-induced colitis model (39). In contrast, Von Hippel-Lindau (VHL)-deficient (*i.e.* Hif1α-stabilized) Treg cells favor Th1 differentiation over Treg differentiation, resulting in colitis development in adoptive T cell transfer model (40) (**Figure 3A**). Besides, HIF signaling pathways plays an important role in the maintenance of the epithelial barrier functions. In an animal colitis model, the systemic administration of Hif1α inhibitor, PX-478, attenuates the protective effects of exogenous H_2_S and epithelial regeneration in dinitrobenzene sulfonic acid-treated rat colitis model (41), whereas Hif1α activation by DMOG (42, 43), FG-4497 (44) or CG-598 (45) results in enhancement of epithelial barrier functions against animal colitis models. Collectively, Hif1α, a master transcription factor driving aerobic glycolysis, regulates T cell differentiation as well as intestinal barrier functions (**Table 1**).

Myc is a proto-oncogene that acts as a transcriptional factor downstream of the mTOR signaling pathway. It is strongly upregulated in both CD4^+^ and CD8^+^ T cells after TCR activation (83, 84). Myc regulates the expression of glycolytic enzymes at both mRNA (84) and protein (83) levels, and affects the expression of lactate transporter (Slc16a1), glycolytic flux (83), and PPP flux (84). Myc also supports the *de novo* pyrimidine/purines synthesis, which are essential for nucleotide production and proliferation in activated T cells (83, 84). Moreover, Myc enhances the expression of amino acid transporter and glutaminolysis in the early phase of TCR activation (4 to 8 hours). Thus, Myc modulates metabolic pathways in CD4^+^ T cells during activation.

Regulatory T cells have a different metabolic signature from conventional effector T cells, and Myc plays a different role in them. Foxp3, the key transcription factor for Treg cells, inhibits c-Myc expression by binding directly to the TATA box of the c-Myc gene (85). In addition, overexpression of Glut1 in Treg cells impairs their functions and exacerbates colitis in a colitis model with adoptive transfer (25) (**Table 1**). However, c-Myc expression is critical for effector Treg generation during early neonatal Treg development (46). Thus, deleting Myc specifically in Foxp3^+^ cells causes early-onset inflammation in multiple organs such as skin, pancreas, liver, lung, and colon in mice (46) (**Figure 3A**). Therefore, the use of Myc inhibitors against proinflammatory T cells should be carefully taken into account for optimal therapeutic effects.

### 2.2 Pyruvate oxidation

Pyruvate can be oxidized into acetyl-CoA, which is a substrate for citrate synthase to generate citrate in the mitochondria (**Figure 2**). Pyruvate oxidation is catalyzed by pyruvate dehydrogenase complex or PDC, which consists of three enzymes: PDHE1, PDHE2 and PDHE3. The activity of PDHE1α is controlled by three phosphorylation sites, Ser232, Ser293 and Ser300, which are tightly regulated by PDC phosphatases (PDPs) and kinases (PDKs). PDC kinase (PDK) is a serine/threonine kinase that inactivates PDC activity by reversible phosphorylation. Therefore, the activity of PDK plays an important role in controlling energy balance and metabolic fitness in cells. PDK has four isoforms (PDK1, PDK2, PDK3 and PDK4). Expression of PDK isoforms is highly enriched in metabolic diseases. Inhibition of PDKs has been suggested as a potential therapeutic target for obesity, diabetes, heart failure, hepatic steatosis and cancer (86, 87). Moreover, the role of PDKs has been investigated in the mitochondrial respiration, activation and polarization of myeloid (88) and lymphoid cells (88).

#### 2.2.1 T cell development and maturation

**Figure 4:**
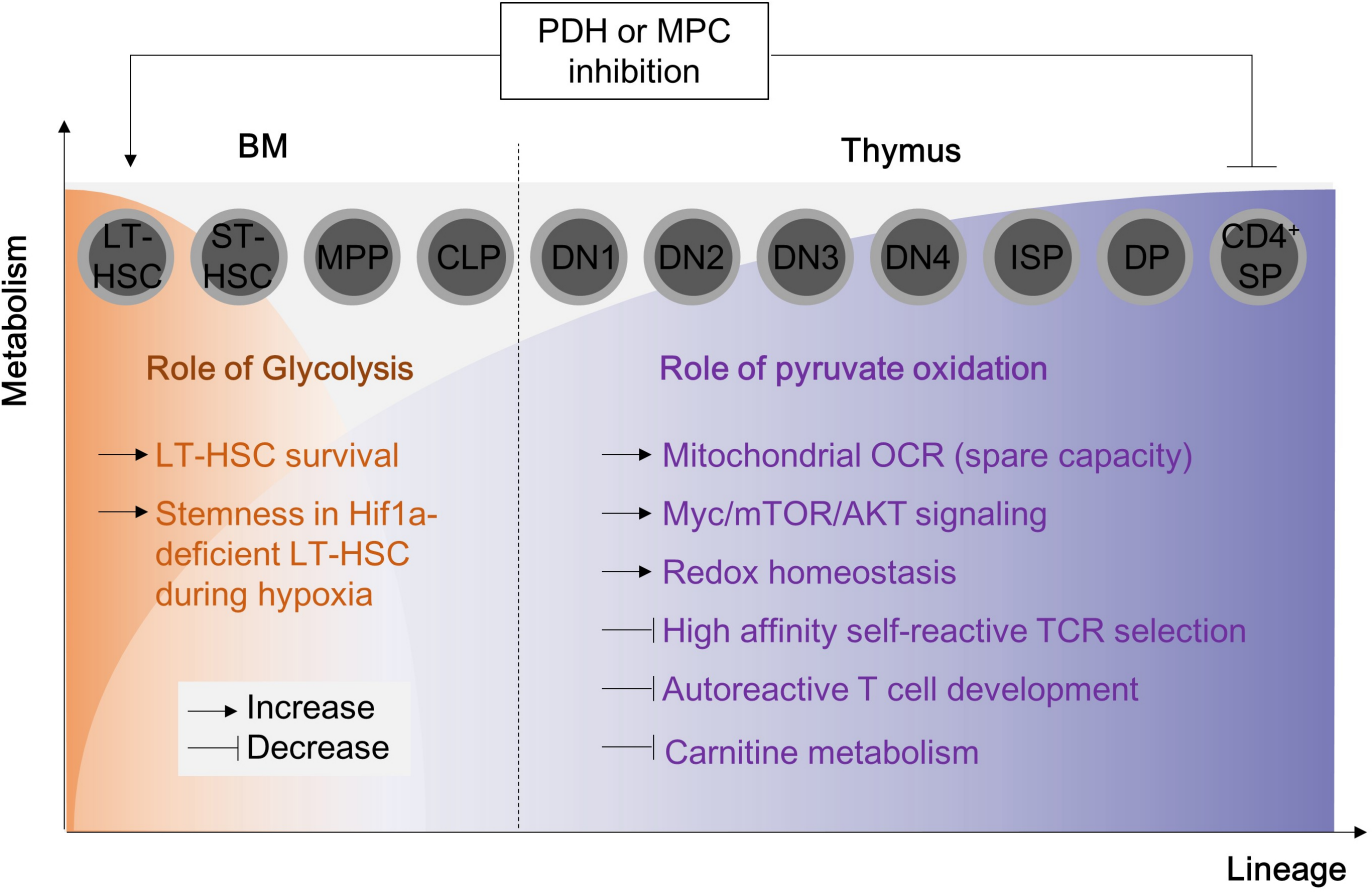
Pyruvate oxidation disruption in hematopoietic stem cells and T cell progenitors alters normal T cell development. The metabolic alterations in hematopoietic stem cells and T cell progenitors can disrupt the normal development of T cells. During early hematopoietic development, pyruvate oxidation is not essential. The double deletion of PDK2/4 has no visible impact on long-term hematopoietic stem cells, but PDH inhibition (for example, with 1-AA) inhibits ST-HSC and MPP cells. Inhibiting MPC1 or PDHE1a, which results in the complete termination of pyruvate oxidation, can lead to severe mitochondrial dysfunction, affecting thymic selection and leading to the development of autoreactive T cells. MPC, mitochondria pyruvate carrier; LT-HSC, long term-hematopoietic stem cell; ST-HSC, short term-hematopoietic stem cell; DN, double negative cells; ISP, immature single-positive cells; DP, double positive cells; SP, single positive cells.

Mitochondrial pyruvate oxidation is a metabolic checkpoint for cell cycle and cellular functions during T cells development. T cells progenitor originate from hematopoietic stem cells (HSC) in the bone marrow. Under physiological conditions in the bone marrow, long-term hematopoietic stem cells (LT-HSC) show a high glycolytic-dependent metabolic profile and impaired oxygen consumption. LT-HSC can preserve their stemness and quiescence by increasing Hif1α-dependent expression of PDKs and reducing mitochondrial mass (89), indicating that pyruvate oxidation is not essential for T cell development at BM. Consistently, defects in PDHA1, PDK2/PDK4 or mitochondrial pyruvate carrier 1 (MPC1) do not affect T cell development at BM (89–91). However, PDK2 or PDK4 knock-in can rescue Hif1α deletion-induced mROS and cell death. Interestingly, only LT-HSC can survive in the *in vitro* cell culture condition with PDH inhibitor, 1-aminoethylphosphinic acid (1-AA), while short-term HSC and multipotent progenitors comes to cell death (89). Thus, pyruvate oxidation is not a requirement for T cell progenitor development at BM (**Figure 4**).

In the later stage, thymocytes mature and differentiate into double negative (DN) T, double positive (DP) T and finally CD4^+^ or CD8^+^ single positive (SP) T cells. During this maturation, T cells metabolically need glucose-derived pyruvate oxidation (90, 91). Blocking pyruvate flux into mitochondria by MPC1 deletion reduces OXPHOS gene expression, cellular oxygen consumption rate, and thymic T cell development (90). PDHE1α deficiency in the thymocytes also affects redox balance and carnitine metabolism (91). Loss of PDHE1α remarkably compromise thymic DP T cell survival and PTEN deletion-induced malignant proliferation in thymocytes (91) (**Figure 4**).

Fully differentiated T cells during homeostasis are largely quiescent and demand a relatively low level of cellular activity. Therefore, they require somewhat higher mitochondrial glucose-derived pyruvate oxidation (65). Stimulation of TCR with CD28 drives expedited metabolic reprogramming through upregulating glycolysis, the pentose phosphate pathway, and glutaminolysis, presumably in the cytosol, yet downregulating pyruvate oxidation (65) in a mitochondrion (84). Certainly, the levels of p-PDHE1 and PDK4, which signify inhibition of pyruvate oxidation, were raised in T cells after early TCR/CD28 activation (13, 49), demonstrating that mitochondrial pyruvate oxidation is essential (**Figure 3B**).

#### 2.2.2 Pyruvate oxidation in T cell responses

In addition to the T cell activation, cellular pyruvate availability also affects T cell differentiation. Ethyl pyruvate (EP) supplementation notably enhances Treg cell proliferation and differentiation in both *in vivo* and *in vitro* (92, 93), whereas Th1 and Th17 differentiation are not affected by EP supplementation *in vitro* (93). Furthermore, the role of pyruvate oxidation in activated CD4^+^ T cells is more important because *in vivo* activated CD8^+^ T cells favor carbon sources from PDH flux (pyruvate oxidation) over pyruvate carboxylase (PC) flux, while *in vitro* activated CD8^+^ T cells prefer PC over PDH flux (94). In addition, CD4-specific deletion of PDHE1α (95) or Glut3 (28) in CD4^+^ T cells compromises glucose-derived acetyl-CoA availability, leading to reduction in the histone acetylation and Th17 polarization (28, 95).

Defeats in mitochondrial pyruvate influx induced by MPC inhibition disrupts mitochondrial calcium homeostasis. This is evident by decreasing carbonyl cyanide 3-chlorophenylhydrazone-induced mitochondrial Ca^2+^ release as well as ATP-induced mitochondrial Ca^2+^ uptake (96). Consequently, nutrient stress by restricted pyruvate oxidation substantially compromises mitochondrial respiration (oxygen consumption rate, OCR) and ATP production and compensatorily enhances autophagy flux (96). This may suggest that mitochondrial pyruvate oxidation sustains mitochondrial health. MPC1 deletion leads to abnormal activation in splenic T cell and results in expansion of auto-reactive T cells in the response to CD3/CD28 stimulation (90) (**Figure 3B**).

In colonic mucosal biopsies from patients with IBD, phosphorylation of PDHE1α, a substrate of PDK4, has been strongly correlated with CD4^+^ T cells (**Figure 3B**). In line with this, the experimental DSS-induced colitis model also displays the significantly upregulated expressions of PDK4 and p-PDHE1α after DSS challenge. Interestingly, CD45^+^ hematopoietic cells including CD4^+^ T cells, neutrophils, macrophages and dendritic cells appears to be a central player in charge with PDK and PDHE1α expression (49) (**Table 2**). Collectively, the restoration of pyruvate oxidation via supplementation of pyruvate or inhibition of PDKs may be directly connected to therapeutic strategy for IBD.

#### 2.2.3 PDH activation through PDK inhibition or PDP activation

Dichloroacetate (DCA) is a structural analogue of pyruvate that inhibits PDKs activity (PDK1 at most among PDKs). Previously, its ability to modulate mitochondrial respiration, ROS generation, and metabolic reprograming has been demonstrated in respect of metabolic syndrome (97, 98), osteoporosis (99) and cancer metabolism (100, 101) as well as immune response (65, 88, 102). In human alloreactive-human peripheral blood mononuclear cell (PBMC), DCA treatment significantly decrease glucose uptake, lactate production, and protein expressions of aerobic glycolysis related enzymes such as Glut1, HK2, LDHA, p-PDH and Myc (103, 104). Furthermore, DCA markedly increased regulatory T cell signature (IL-10 secretion and Foxp3 protein expression), and yet decreased effector T cell signature (such as T-bet, GATA3, and RORγT expression) in alloreactive human PBMC. These data suggests that PDK blocker, DCA, mitigate aerobic glycolysis and favor differentiation toward immunosuppressive regulatory T cell rather than proinflammatory effector T cells (103, 104) (**Figure 3B**). In line with this observation, activated CD4^+^ T cells (isolated from BALB/c normal mice) by CD3/CD28-mediated TCR stimulation had decreased lactate production and effector cytokine productions (IFN-γ, IL-5, and IL-17) in response to DCA for 48 hours (105) (**Figure 3B**).

Under hypoxia, both glycolysis and pyruvate oxidation are critical for the survival of effector memory (EM) T cells Glucose deprivation or 2-DG treatment significantly reduces mitochondrial membrane potentials and consequently induces apoptosis in EM T cells. However, the addition of sodium pyruvate or DCA dramatically restores 2DG-induced mitochondrial membrane potential declines and enhances survival under hypoxic stress (106). This finding suggests that glucose oxidation via pyruvate metabolism stabilizes mitochondrial membrane potentials and thereby increases survivability under hypoxic conditions.

PDK also modulates CD4^+^ T cell differentiation. First, its pharmacological inhibitor, DCA, has been clearly demonstrated to induce Treg differentiation and suppress Th17 differentiation (48, 107, 108) in part through ROS generation (**Figure 3B**). However, the current understanding of the mechanisms by which PDK inhibitors regulate T cell differentiation is contradictory. The predominant isoforms of PDKs in naïve, Th17 and regulatory T cells are PDK1 and PDK3 (48, 107). One paper shows that PDK1 deficiency, the strongest target isoform among PDKs that is possibly inhibited by DCA, compromises Th17 differentiation and accelerates Treg differentiation (48), which suggests that the effects of DCA are dependent on PDK –presumably PDK1- activity. On the other hand, it has been shown that the immunosuppressive effects of PDK inhibitors were ironically independent of PDKs because the effect of DCA on manipulating T cell differentiation is persistent even under the knockdown of PDK1 and PDK3 (107). This may suggest that the anti-inflammatory effects of DCA are likely followed through the inhibition of PDK2 and PDK4, or unidentified non-canonical pathways such as ROS production (107). Despite the differences in methods of isolation and differentiations of naïve CD4^+^ T cells that were used in those papers, PDK activity is undoubtedly pivotal in T cell differentiation. In addition, PDK1 (60) or PDK4 (49) knockdown significantly reduces IL-17 secretion in *in vitro*-polarized Th17 cells (**Figure 3B**).

The effects of PDK inhibition on Th1 and Th2 differentiation have not been thoroughly investigated. DCA treatment significantly enhances IFN-γ secretion and production in splenocytes (109) as well as Th1-polarized CD4^+^ T cells (108), indicating enhanced Th1 differentiation. In contrast, one report showed that DCA treatment fails to reduce Th1 differentiation and effector function (48).

PDK1 has been implicated in the early stages of TCR signaling pathways during T cell activation, in addition to its role in the mitochondrial matrix (**Figure 3B**). In PA-R CD8^+^ T cells, TCR activation by CD3 results in the downstream signaling complex, including ZAP70 and Lck, which binds and activates PDK1 within a few minutes (65). Consequently, due to the impediment of mitochondrial pyruvate influx, the cumulative cytosolic pyruvate allows an augmentation in lactate biosynthesis. In this way, the TCR complex rapidly utilizes aerobic glycolysis during the activation process (65, 110). In summary, these data suggest that inhibition of PDKs reduces anaerobic glycolysis, improves mitochondrial function, promotes ROS production, decreases Th17 differentiation, and increases immunosuppressive Treg function. Therefore, targeting pyruvate oxidation flux to manipulate T cell functions is a promising strategy in the treatment of inflammatory bowel disease (IBD).

Notably, EP has been shown to significantly increase the population of CD4^+^CD25^+^Foxp3^+^ regulatory T cells in both peritoneal cells, lamina propria, and the Peyer’s patches (92) (**Figure 3B**). Furthermore, EP treatment attenuates Th17 cell infiltration in the intestine and prevents trinitrobenzene sulphonic acid (TNBS)-induced colitis in mice (47). *In vivo* DCA treatment (2g/L, *ad libitum*) significantly reduces CD4^+^ T cell accumulation, especially Th17 cells, in the spleen and mesenteric lymph nodes in a naive T cell adoptive transfer colitis model (48). Unfortunately, DCA treatment fails to prevent intestinal inflammation due to the non-responsive Th1 cells to DCA. However, PDK inhibition by DCA successfully attenuates EAE progression, which is another Th17-mediated neuronal disease (48). Although DCA treatment is an efficient way to restrain the development of Th17-mediated inflammation, other approaches inhibiting PDKs may be warranted.

Recently, we have reported the pathological role of PDK4 in CD4^+^ T cell (49, 91). PDK4 is highly expressed during early T cell activation, and its deletion leads to the suppression of aerobic glycolysis. Certainly, PDK4 KO mice were found to be more resistant to DSS-induced colitis. In addition, CD4^+^ T cells deficient in PDK4 induce less intestinal inflammation in both DSS-induced colitis and naïve T cell adoptive transfer colitis. Furthermore, treatment with the pharmacological PDK4 inhibitor, GM-10395, compromises CD4^+^ T cell activation and attenuates DSS-induced colitis (49) (**Table 2**).

Pyruvate dehydrogenase phosphatase (PDP) is a mitochondrial matrix enzyme that sustains PDC activity. PDP1 is predominantly expressed in mitochondria from skeletal muscle, whereas the PDP catalytic subunit 2 (PDP2) is expressed in the liver and many other cells, including white blood cells. In agreement with the effects of DCA on T cell differentiation, PDP2 overexpression, which enhances PDC and pyruvate oxidation, inhibits glycolysis and Th17 differentiation (111) (**Figure 3B**).

### 2.3 Glutaminolysis

Glutaminolysis is a catabolic mechanism by which the amino acid glutamine is degraded to glutamate, α-ketoglutarate (α-KG), aspartate, and pyruvate (**Figure 3C**). Glutamine is initially catalyzed by mitochondrial glutaminase (GLS) to produce glutamate, which is an essential amino acid playing various roles in cellular physiology. Glutamate can be further oxidized by glutamate dehydrogenase (GDH), glutamic oxaloacetic transaminase (GOT), and glutamic pyruvic transaminase (GPT) to produce α-KG, which supports TCA cycle intermediate via an anaplerotic route for mitochondrial ATP generation and a substrate for histone/DNA methylation during epigenetic modifications (**Figure 3C**). Particularly, the glutamate-α-KG cycle plays the most important role in cancer cells or other proliferating cells to maintain nitrogen metabolism. α-KG gathers nitrogen atoms from excess amino acids, while glutamine may contribute nitrogen atoms to synthesize nucleotides or non-essential amino acids to support cellular proliferation, protein synthesis, and nucleotide synthesis (**Figure 3C**). Thus, a therapeutic strategy targeting glutaminolysis could be a superior resolution to make not only cancer cells but also inflammatory, proliferating CD4^+^ T cells vulnerable.

#### 2.3.1 Glutamine availability

In activated T cells, glutaminolysis plays a vital role in their proliferation and effector functions. In mouse splenocytes or T cells, removal of glutamine completely blocks their effector functions (i.e., proliferation and secretion of IL-2 and IFN-γ (112)) in spite of comparable expression of activation cell surface marker (CD25, CD69, and CD98 (112)). This dependence of glutamine for proliferation of activated splenocytes or PBMC cannot be replaced by supplementation of glutamate, α-KG, asparagine, or proline, which are substrates that can substitute for glutamine (112, 113).

In addition, activated human CD4^+^ T cell for 24 hours do not utilize glucose or glutamine as a carbon source to fuel mitochondrial respiration, as the pharmacological treatment using MPC inhibitor (UK5099), GLS inhibitor (CB839 or BPTES), or a combination of both fail to decrease mitochondrial oxygen consumption (52, 114). However, glutamine deprivation reduces mitochondrial oxygen consumption and ATP production (51) (**Figure 3C**).

Moreover, glutamine availability is still critical in the differentiation of naïve CD4^+^ T cells. Glutamine deprivation leads to the differentiation of naïve CD4^+^ T cells into Foxp3^+^ regulatory T cells with robust *in vivo* proliferative potentials and immune suppressive effects (51, 113) and a reduction in effector cytokine production such as IFN-γ and IL-17A (52) (**Figure 3C**). Strikingly, the skewing of naïve CD4^+^ T cells to Foxp3^+^ Tregs with glutamine restriction cannot be reversed by glutamate or α-KG supplementation, indicating that glutamine is not required for carbon source (113).

Moreover, naïve CD4^+^ T cells grown with low glutamine availability, even under Th1 or Th17-polarizing conditions, favor the differentiation into Foxp3^+^ Tregs while blocking the differentiation into Th1 and Th17 cells (51, 52), respectively (**Figure 3C**). These observations suggest that glutamine itself, not those intermediates of glutaminolysis such as glutamate and α-KG, plays an essential role in modulating effector functions, mitochondrial ATP synthesis, and differentiation in CD4^+^ T cells.

*De novo* nucleotide synthesis requires glutamine-driven aspartate as a source of nitrogen atoms for the formation of the purine/pyridine ring (115). It is well established that aspartate biosynthesis from glutamate via GOT1/2 is required for proliferation in mammalian cells (116, 117). In CD4^+^ T cells, inhibition of *de novo* purine/pyrimidine synthesis by N-phosphonacetyl-l-aspartate (PALA) and mycophenolic acid (MPA) promotes the differentiation of Foxp3^+^ Tregs, which is consistent with the effects of glutamine deprivation (**Figure 3C**). In addition, the generation of Foxp3^+^ Tregs is abolished under low glutamine conditions when glutamine synthetase (GS), which synthesizes endogenous glutamine from glutamate, is inhibited by Methionine sulfoximine (MSO) (113) (**Figure 3C**). These findings suggest that the nitrogen atoms in the amide group of glutamine are primarily used for nucleotide synthesis, rather than the carbon backbone of glutamine, in proliferating CD4^+^ T cells.

Despite that glutamine supplementation attenuates intestinal inflammation in the DSS-induced colitis animal model (50) (**Table 2**), Foxp3^+^ T cells induced by glutamine restriction have shown superior immunosuppressive capacity *in vivo* to prevent IBD using the adoptive T cell transfer mouse model. Injection of either CD4^+^Foxp3^+^ natural Tregs or Foxp3^+^ T cells grown under glutamine-limited conditions fully protects against colonic infiltration of immune cells, weight loss, and activation of effector T cells. Moreover, glutamine withdrawal has been shown to enhance the proliferation of Foxp3^+^ T cell *in vivo* (51) (**Figure 3C**).

#### 2.3.2 Glutaminase

GLS enzyme activity and mRNA expression are highly induced during early T cell activation (112, 118). However, GLS activity is considerably lower than the activity of GOT or GDH in T cells (112). This indicates that GLS, which is the first enzymes in the process of deamidation of glutamine, is likely to be a limiting factor in T cell activation and presumably T cell differentiation. Under Th0 activating condition by CD3 and CD28 stimulation, GLS inhibition by compound 968 or BPTES significantly impairs the expression of T cell activation marker (CD25 and CD226), secretion of effector cytokines such as IFN-γ, TNF-α, IL-2, and IL-17 and chemokine receptors (CCR6 and CXCR3) (118). Nevertheless, the mode of action by GLS inhibition is quite astonishingly different from that by glutamine deprivation on T cell differentiation. Treatment with GLS inhibitors (CB839 or BPTES) significantly decreases proliferation, IL-17 cytokine production (52, 119) and ATP-coupled OCR (120) in Th17 cells but increases proliferation and IFN-γ production in Th1 cells with an enhanced exhausted phenotype (i.e. PD-1, Lag3, and Tim3) (52) (**Figure 3C**). Moreover, the genetic deletion of GLS leads to the attenuation of RORγt expression in Th17 cells but the accumulation of t-bet expression in Th1 cells while not affecting Foxp3 expression in Treg (**Figure 3C**). In addition, the generation of α-KG decreases in CB839-treated Th1 but not Th17 cells, whereas 2-HG increased in both Th1 and Th17 (**Figure 3C**). Given that α-KG is an important cofactor for both histone and DNA demethylation, GLS deficiency may influence T cell differentiation mechanistically through not only TCA anaplerotic intermediate but also alteration of epigenetic modifications. CB839-treated Th1 cells display decreased global H3K27 trimethylation and more Th1 related genes such as *Ifng* with opened chromatin accessibility, and supplementation of a-KG reverses the decreased global methylation and opened chromatin status in CB839-treated Th1 cells (52) (**Figure 3C**). Of note, α-KG has been previously reported to regulate IL-2-sensitive effector gene expression in Th1 cells through the association of CCCTC-binding factor in part (121). Taken together, it is suggested that GLS deficiency has distinct mechanisms of differentiation of Th1 and Th17 cells. Patients with CD possess significantly higher numbers of GLS1-positive cells in the inflamed regions of the lamina propria than the control patients (53). Collectively, targeting GLS may impair the immune responses of the infiltrated T cell, particularly Th17 *in vivo* for treating IBD. Adoptive transfer of GLS-deficient naive CD4^+^ T cell to Rag1 KO mice indeed fails to induce weight loss and intestinal inflammation (52) (**Figure 3C**). Treatment of BPTES also effectively ameliorates spontaneous intestinal inflammation in IL-10 deficient mice by restoring Th17/Treg balance (53) (**Figure 3C**; **Table 2**).

Metabolic adaptations has emerged as a critical process in T cell activation, survival, and differentiation. Manipulating the metabolism of T cells can be promising strategies for treating IBD. However, metabolic modulators may have unwanted effects on not just only pathogenic T cells, but also intestinal epithelial cells, endocrine system, bile acid production, and intestinal microbial metabolism to some extent. Thus, further studies are required.

## 3 Targeting T cell mitochondria for treating colitis

### 3.1 Mitochondrial biogenesis

**Figure 5:**
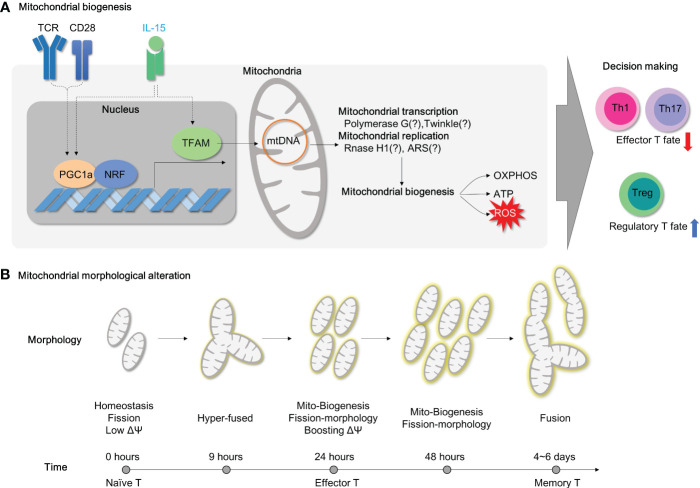
Mitochondrial biogenesis and morphology changes during T cell activation. **(A)** For T cell activation, it is necessary to undergo mitochondrial biogenesis to meet the energy requirements of the cell. When TFAM is inhibited, energy homeostasis is disrupted, and this results in the polarization of effector T cells. Additionally, PGC1a/TFAM activation is stimulated by IL-15, which leads to mitochondrial biogenesis. **(B)** The morphology of mitochondria in CD4^+^ T cells changes during TCR-mediated activation as time progresses. TCR, T cell receptor;PGC1a, peroxisome proliferator-activated receptor-gamma coactivator 1; NRF, Nuclear Respiratory Factor; TFAM, transcriptional factor A mitochondrial; OXPHOS, oxidative phosphorylation; ATP, Adenosine triphosphate; ROS, Reactive oxygen species; ARS, aminoacyl tRNA synthetase.

Mitochondrial biogenesis is a fundamental process for supplying sufficient energy demands during inflammation in CD4^+^ T cells. In quiescent circulating CD4^+^ T cells, the expression levels of glycolytic enzymes (such as HK2, Pyruvate kinase M2, LDHA) and the basal glycolysis rate determined by extracellular acidification rate are relatively higher than those in CD8^+^ T cells (122). At the same time, unstimulated CD4^+^ T cells possess a higher number of mitochondrial masses than CD8^+^ T cells despite maintaining mitochondrial respiration at the same level, suggesting that quiescent CD4^+^ T cells stock a decent amount of mitochondrial biogenesis for later use (122). Indeed, TCR stimulation rapidly drives mitochondrial reworking in CD4^+^ T cells. Upon activation by TCR ligation, splenic T cells increase mitochondrial membrane potentials after 12 hours and robust mitochondrial biogenesis (mtDNA and mitochondrial mass) after 48 hours (123) (**Figure 5B**). In accordance with splenic T cell activation, the mitochondria in activated CD4^+^ T cells turn into hyper-fused form after 9 hours and become highly energetic with re-fragmentation of mitochondrial morphology, enhanced mitochondrial biogenesis, and accumulated TCA intermediates after 24-hour exposure to CD3/CD28 antibodies (124) (**Figure 5B**). During this early activation, T cells accelerate the fatty acid biosynthesis and uptake process (125). Meanwhile, mitochondria rearrange one-carbon metabolism within mitochondria, which enhances redox homeostasis and promotes *de novo* purine biosynthesis (124). After prolonged activation (more than 96 hours), CD4^+^ T cells continuously increase mitochondrial biogenesis and respiration without mROS-induced mitochondrial dysfunction (126) (**Figure 5B**). In addition to activation, mitochondrial biogenesis and fitness also orchestrate immune-senescence in CD4^+^ T cells during aging (127). Therefore, controlling mitochondrial biogenesis could provide therapeutic opportunities in resolving CD4^+^ T cell-mediated inflammation.

Mitochondrial biogenesis is primarily regulated by the nuclear transcription factor peroxisome proliferator-activated receptor γ coactivator‐1 (PGC‐1) family of transcription coactivators, which includes PGC1α, PGC1β, and PPRC1 (**Figure 5A**). PGC1α, together with NRF-1, initiates the transcription of various mitochondrial proteins, including mitochondrial transcription factor A (TFAM), which is then imported into mitochondria (128). Among mitochondrial nucleoids, TFAM is the most abundant DNA-binding protein, which functions in mitochondrial DNA stability and replication. Notably, Tfam mRNA or protein expressions are remarkably upregulated in CD4^+^ T cells after 4- or 24-hour exposure to CD3/CD28 stimulation, respectively (129) in line with mitochondrial biogenesis.

#### 3.1.1 Mitochondrial transcription factor A

Deficiency of TFAM in CD4^+^ T cells leads to mitochondrial dysfunction characterized by reduced mtDNA copy number, decreased transcription of mitochondrial proteins, and impaired fatty acid oxidation, despite compensatory increases in mitochondrial mass. Strikingly, Tfam-deficient CD4^+^ T cells also have dysfunctions in transcription factor EB (TFEB)-induced endolysosomal biogenesis, impaired autophagic flux, and abnormal accumulation of lipids such as sphingomyelin and triglycerides (14). Furthermore, the mitochondrial dysfunctions induced by Tfam deficiency lead to effector T cell differentiation favoring Th1 cell differentiation under Th1 or Th2 polarizing conditions, as well as more pathogenic IFN-γ-secreting Th17 cells (14). Consistent with this, Tfam deletion in regulatory T cells induces mROS and switches metabolism towards glycolysis (54). Tregs in the absence of Tfam have significantly reduced Foxp3 expression, which is indispensable for sustaining immune-tolerance (54). Moreover, Tfam deficiency leads to the development of more proinflammatory IFN-γ (54), IL-13 or IL-17a secreting Tregs (54) (**Figure 5A**). Collectively, mitochondrial Tfam in CD4^+^ T cells governs mitochondrial fitness, the balance between effector and regulatory T cells, and autoimmunity. Therefore, it is suggested that Tfam deficiency in CD4^+^ T cells is likely to prompt the development of autoimmune diseases such as IBD. Undoubtedly, CD4-specific Tfam KO mice are more susceptible to chemical-induced experimental colitis model using 3% DSS (14) (**Table 3**). Of note, CD4-specific Tfam-deficient mice over 2 months of age exhibit premature aging syndromes with multiple organ dysfunctions associated with aging (130). Moreover, the infusions of Tfam-deficient Tregs functionally fail to resolve the accumulation of pathogenic CD4^+^ effector T cells in the adoptive T cell transfer colitis model and accordingly advance disease severity (54) (**Figure 5A**; **Table 3**).

In addition to TFAM, there are more gene sets related to mitochondrial biogenesis regarding mitochondrial DNA replication (e.g., Polymerase G and Twinkle) and mitochondrial transcription (e.g., RNase H1 and mitochondrial aminoacyl-tRNA synthetase) (131, 132). Yet, none of them have been thoroughly elucidated in mitochondrial functions and T cell responses. Recently, one paper shows that T cells with *sti* mutation (defects in alanyl-tRNA synthetase) have compromised TCR signal initiation machinery in T cells (133). Therefore, targeting mitochondrial biogenesis is still considered a viable option for IBD therapy and new developments in this field are expected to appear in the near future.

#### 3.1.2 Interleukin-15

IL-15 is a cytokine known for T cell homeostasis, generation of memory T cells and prevention from cell death (134–136). Nevertheless, IL-15 has been recognized as a critical cytokine in mitochondrial biogenesis in T cells. IL-15-treated CD8^+^ T cells have elongated hyper-fused mitochondria with increased mitochondrial mass (137, 138) and TFAM expression (139) compared to unstimulated or IL-2 treated CD8^+^ T cells. In addition, IL-15 has also been shown to play a central role in CD4^+^ T cell proliferation (140), Th17 effector function (141), T helper cell differentiation (142), and neuronal-autoimmunity (141). Moreover, IL-15 remarkably restored augmented mitochondrial biogenesis (TFAM and PGC1α expression) (**Figure 5A**), diminished mitochondrial mass (mitotracker-green staining), and impaired proliferation in cycling Tregs of immune non-responder subjects after HIV infection (143). In addition, deprivation of the IL-15 cytokine presumably shifts immunosuppressive Foxp3-positive cells to proinflammatory RORγt-positive cells (55). Collectively, it is highly affirmative that IL-15 has a possible implication in controlling mitochondrial biogenesis in CD4^+^ T cells and its therapeutic applications in IBD. Although genetic deletion of IL-15 (i.e. using IL-15 whole-body KO mice) is not successful in treating DSS-induced colitis model in mice (144) (**Table 3**), adoptive transfer of B6 CD4^+^ T cells to IL-15-deficient Rag KO mice induces colonic inflammation compared to the control, and adoptive transfer of IL-15 receptor-deficient Treg fails to suppress severe colonic inflammations with extensive mucosa damage in CD4^+^ T cell transfer colitis model (55) (**Table 3**), suggesting a direct role of IL-15 in colitogenic CD4^+^ T cells.

### 3.2 Mitochondrial oxidative phosphorylation

Mitochondria produces the bioenergetics molecule ATP from NADH and FADH_2_ by oxidative phosphorylation via complex I, II, III, IV, and V. As expanding mitochondrial respiration is a critical progression during early T cell activation, as mentioned above, inhibition of mitochondrial respiratory complexes might be helpful in buffering T cell immune responses (145) and treating T cell–mediated inflammation such as IBD.

**Figure 6:**
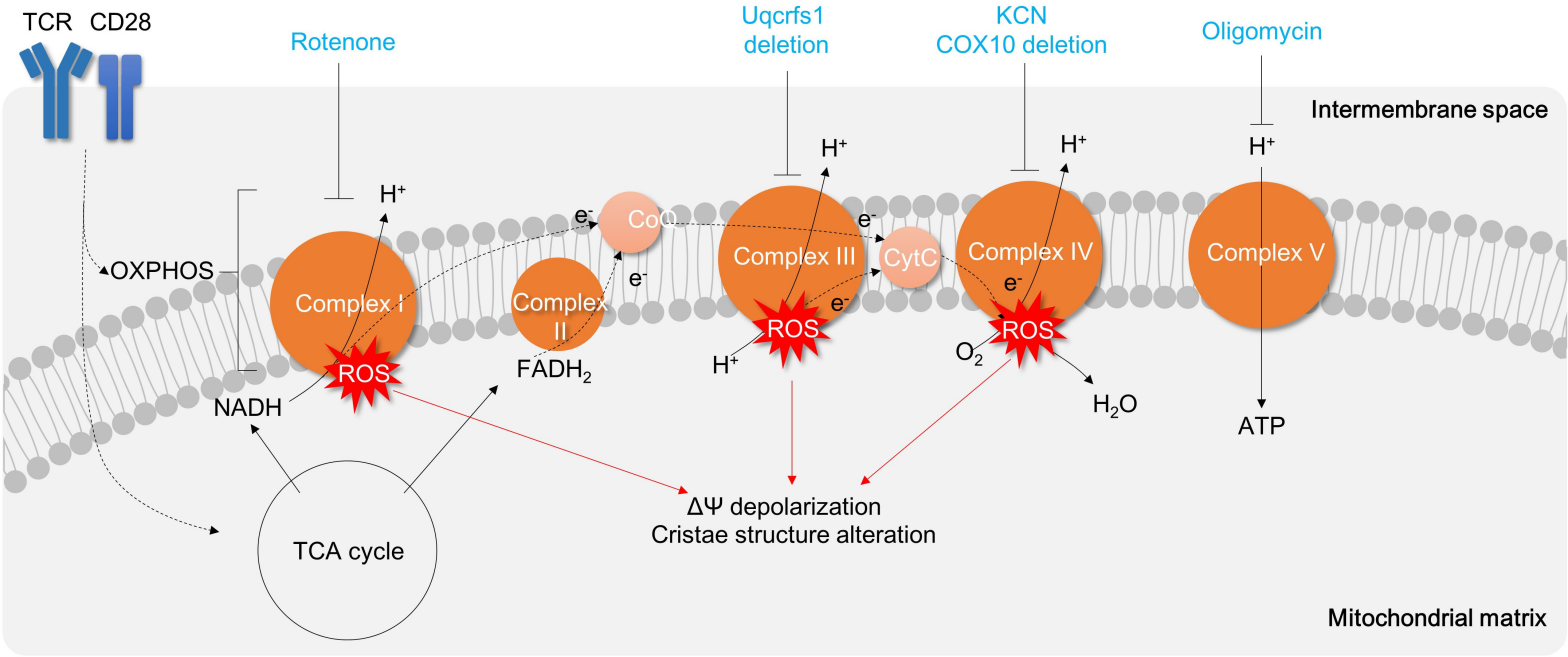
Targeting Mitochondrial Respiration Complexes for Modulating T Cell Activation and Inflammation. Activation of T cells results in upregulation of the TCA cycle and OXPHOS, which leads to the generation of oxidative stress. Inhibition of Complex V (such as oligomycin) hinders mitochondrial respiration and subsequently suppresses T cell activation and proliferation. Other mitochondrial respiratory complexes are also being explored as potential targets for drugs aimed at reducing T cell inflammation. TCR, T cell receptor; OXPHOS, oxidative phosphorylation; TCA cycle, tricarboxylic acid cycle;ROS, reactive oxygen species; CytC, Cytochrome C.

T cell metabolism is strongly influenced by the surrounding microenvironment, including nutrient (146) and oxygen availability (147, 148). As a result, the distinct *in vivo* conditions (characterized by relatively high lactate and low oxygen levels) and *in vitro* conditions (with high glucose and high oxygen levels) inherently lead to different functionalities of matured T cells. Specifically, *in vivo*-differentiated Th17 cells exhibit a higher dependence on OXPHOS, whereas *in vitro*-differentiated Th17 cells rely more on aerobic glycolysis. Consequently, *in vivo*-differentiated Th17 cells appear to be more sensitive to complex V inhibitor, oligomycin, treatment (60). In line with this, oligomycin treatment significantly reduces Th1 proliferation (149), IL-17α secreting CD3^+^ T cells, and thereby attenuates TNBS-induced colitis animal model comparable to the group treated with RORγt inhibitor ursolic acid (60) (**Figure 6**; **Table 3**).

Other mitochondrial respiratory chain complexes I, III, or IV are also critical in T cell activation and differentiation. Treatment of rotenone, a complex I inhibitor, significantly attenuates Th1 polarization in CD4^+^ T cells (149) (**Figure 6**). Uqcrfs1, a subunit of complex III, is required to promote excessive mROS production and IL-2 cytokine secretion in CD4^+^ T cells (66) (**Figure 6**). Moreover, the inhibition of complex IV by CD4^+^ T cell-specific deletion of COX10 or treatment of complex IV inhibitor potassium cyanide impairs mitochondrial respiration, mitochondrial cristae structure, T cell activation, proliferation, Th1 (13) and Th17 differentiation and results in apoptosis (13, 150) (**Figure 6**). Therefore, mitochondrial oxidative respiration is critical in T cell activation, differentiation, and effector functions, and targeting mitochondrial oxidative phosphorylation against IBD may have therapeutic benefits.

### 3.3 Mitochondrial calcium homeostasis

T cell activation triggers calcium influx that orchestrates approximately three-quarters of the transcription associated with cell cycle, signal transduction, apoptosis, and effector function (151). Previous literature has utilized blocking or modulating plasma membrane calcium channels such as transient receptor potential ankyrin (TRPA), transient receptor potential vanilloid (TRPV), calcium release-activated calcium modulator (ORAI), and calcium-activated potassium channel (KCa) in mouse colitis models (152–161). Moreover, immunosuppressive drugs, cyclosporine A (162) or FK506 (163), classical inhibitors of calcium-dependent calcineurin, induce clinical responses in patients with IBD. In addition, calcium response in human CD45RO^+^ CD4^+^ central/effector memory T cells is greater than in CD45RA^+^ CD4^+^ naïve T cells (164). While it is strongly predictable that manipulating calcium signaling in T cells could have therapeutic applications, it has yet to be proven whether maintaining mitochondrial calcium homeostasis in T cells has any therapeutic benefit for treating IBD.

**Figure 7:**
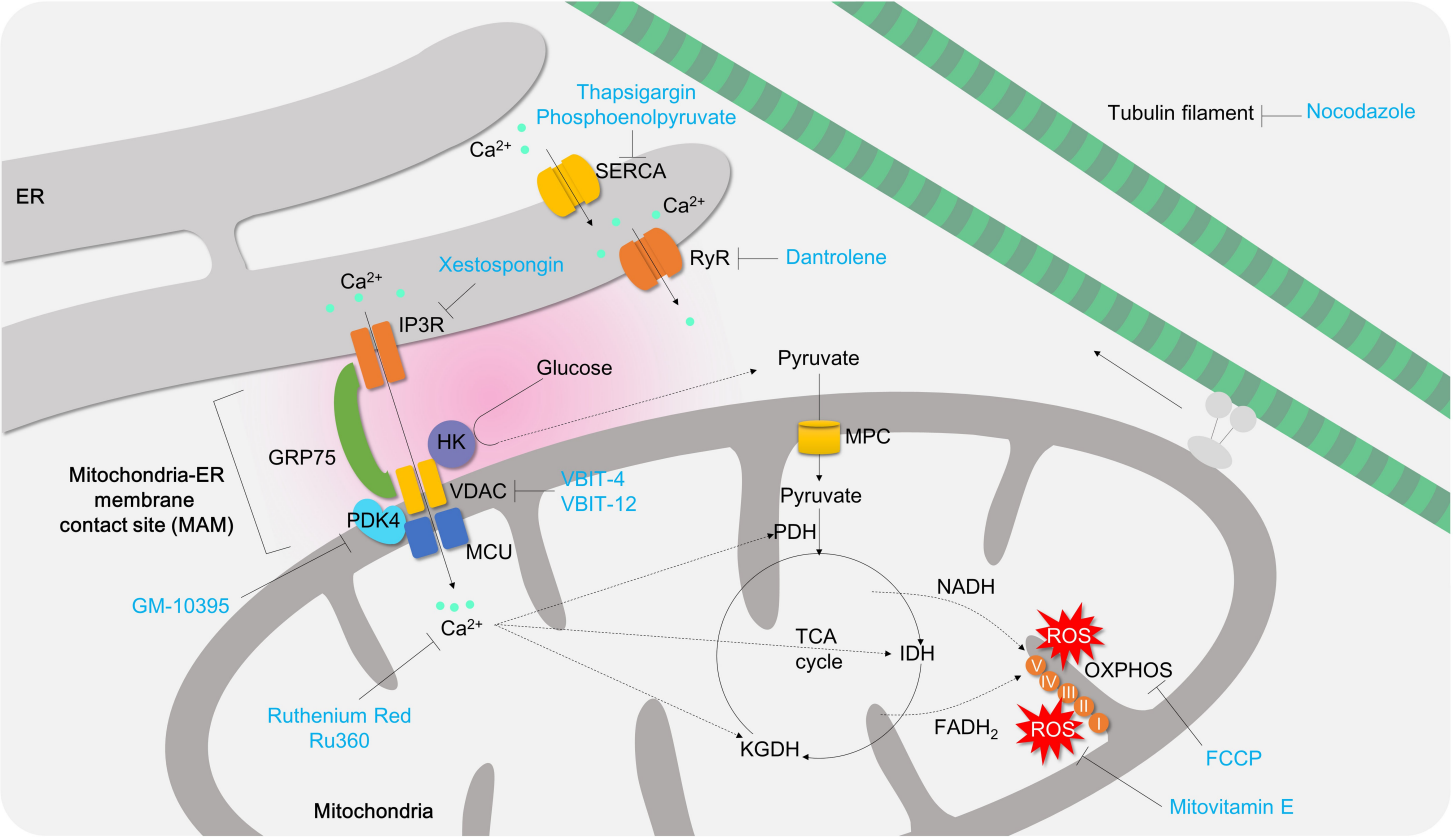
The role of mitochondrial calcium signaling and MAM in controlling T cell activation and differentiation. Under normal physiological conditions, mitochondria play a critical role in maintaining redox balance and energy metabolism by absorbing local calcium ions. However, during T cell activation, mitochondria take up cytosolic or ER calcium through the mitochondria-ER associated membrane contact site (MAM), which promotes TCA cycle, OXPHOS, and oxidative stress production. If mitochondrial calcium is depleted (e.g., using Ru360 or Ruthenium Red), or MAM formation is inhibited (e.g. using GM-10395, Xestospongin, VBIT-4, VBIT-12, nocodazole), it can suppress T cell activation, proliferation, and differentiation into effector T cells. ER, endoplasmic reticulum; SERCA, sarco/endoplasmic reticulum Ca^2+^-ATPase;IP3R, inositol 1,4,5-trisphosphate receptor;HK, hexokinase;GRP75, glucose-regulated protein 75;PDK4, pyruvate dehydrogenase kinase 4; PDH, pyruvate dehydrogenase; MPC, mitochondria pyruvate carrier; VDAC, voltage-dependent anion channel; RyR, Ryanodine receptor; FCCP, Carbonyl cyanide 4-(trifluoromethoxy)phenylhydrazone; IDH, isocitrate dehydrogenase;KGDH, α-ketoglutarate dehydrogenase.

Mitochondria have been reported to relocate to the immune synapse (IS) in T cells at early stage of T cell activation (165). When T cells encounter antigen-presenting cells, they require a large quantity of ATP to maintain immunological synapse (IS) architecture as a cellular substrate for immune activities such as kinase action, motor proteins, and degranulation. In addition, the mitochondria at the IS support robust calcium channel opening at the plasma membrane by clearing excess calcium, and thus amplify the calcium signaling pathway in T cells (166). Nonetheless, this mitochondrial calcium governs mitochondrial ATP and mROS production (167) (**Figure 7**). Three dehydrogenase enzymes consisting of TCA-cycle (PDH, α-KG dehydrogenase (KGDH), and isocitrate dehydrogenase (IDH)) are well-known enzymes directly regulated by mitochondrial calcium (168–170) (**Figure 7**). In some cases, mitochondrial calcium plays a role as a weak uncoupler, perhaps due to the pH gradient (ΔpH) and membrane potentials (ΔΨ) across the inner membrane (167). The production of mROS and the maintenance of mitochondrial membrane potential are essential for effector function at the early T cell activation. Treatment of mitochondrial-targeted antioxidant mitovitamin E or mitochondrial membrane potential depolarizing agent, carbonyl cyanide 4-(trifluoromethoxy)phenylhydrazone (FCCP), successfully attenuates IL-2 secretion in CD4^+^ T cells (66) (**Figure 7**). Thus, mitochondrial calcium unquestionably plays an important role in regulating T cell activation and their effector functions.

Mitochondrial calcium instability is contributed by endoplasmic reticulum (ER) calcium deficiency, which transports approximately 25 to 50 percent of ER calcium release triggered by caffeine or thapsigargin to nearby mitochondria in mast cells or myocytes (171). There are two options to control ER calcium for mitochondrial calcium homeostasis: (i) inhibiting ER calcium importer to limit the ER-Ca^2+^ pool or (ii) inhibiting ER calcium exporter to mitochondria. However, the former approach may not be helpful to treat colitis. Sarco/endoplasmic Reticulum Calcium ATPase (SERCA) is a calcium ATPase which transfers the cytosol calcium into ER. Thapsigargin, an irreversible SERCA inhibitor, is known to cause ER calcium shortage and markedly enhance Th17 differentiation, likely due to ER stress (172) and amplified Ca^2+^/NFAT signaling (64). Non-canonical SERCA inhibitor phosphoenolpyruvate augments Ca2^+^/NFAT signaling in Th1 cells as well (64). Furthermore, ER stress and unfolded protein responses have been associated with IBD (173). Therefore, mitochondrial calcium controlling requires other strategies without alleviating ER stress.

Ryanodine receptor (RyR) and IP3R mediate calcium release from ER to cytosol or mitochondria. Inhibition of RyR by dantrolene or IP3R by xestospongin C has been shown to restore thapsigargin-induced ER stress in hepatocytes (174) or islet cells (175) (**Figure 7**). Although there are no animal colitis studies or clinical trials conducted to date, the applications of these inhibitors for colitis might be worth a try. In T cells, RyR inhibition by ryanodine or dantrolene successfully attenuates store-operated calcium entry (SOCE), T cell proliferation and IL-2 production (176, 177) (**Figure 7**). IP3R inhibition by xestospongin significantly inhibits differentiation of naive T cells to pro-inflammatory Th9 cells (178) (**Figure 7**).

However, targeting mitochondrial calcium is also a promising approach. Treatment of mitochondrial calcium uniporter blockers, Ru360 and Ruthenium red (179), significantly inhibits mROS production in CD4^+^ T cells (66) and IFN secretion and cell proliferation (180). In *in vivo* TNBS-induced colitis animal model treatment of ruthenium red remarkably attenuates intestinal inflammations. Therefore, targeting mitochondrial calcium is sufficient to decrease T cell effector function and intestinal inflammation (56) (**Figure 7**; **Table 3**).

#### 3.3.1 Mitochondrial calcium uniporter

Mitochondria contain a number of calcium transport channels, including the mitochondrial calcium uniporter (MCU), which is sub-localized in the inner membrane of mitochondria (181). Mitochondrial-mediated Ca^2+^ uptake plays an important role in cytosolic Ca^2+^ buffering in mast cells. Studies have shown that in MCU-deficient mast cells, antigen-induced beta-hexominidase release was suppressed, suggesting a role for MCU-mediated mitochondrial Ca^2+^ flux in mast cell degranulation (182). Furthermore, knockdown of mitochondrial MCU has been shown to reduce cytoplasmic calcium fluctuation and SOCE in mast cells (183). However, the pathological role of both MCU and MICU1 (a gatekeeper of MCU-mediated mitochondrial Ca^2+^ uptake) in T cells remains poorly understood. Loss of MICU1 has been shown to generate excessive ROS, delay proliferation, and impair migration in HeLa cells (184), Thus, blocking MCU or MICU1 in CD4^+^ T cells is likely to result in mitochondrial Ca^2+^ instability and ROS generation, leading to suppression of cell proliferation and gut penetration in pathogenic CD4^+^ T cells (**Figure 7**).

#### 3.3.2 Voltage-dependent anion channel

Voltage-dependent anion channel (VDAC) is a mitochondrial outer membrane transporter, and has been associated with mitochondrial calcium transport, cell death, and lupus-like autoimmunity (185). Upon 24-hour T cell activation, VDAC1 expression increases along with the Glut1 and HK2 in PBMC (186), which are activation signatures of T cells. In addition, PBMC from patients with coronavirus-A displays mitochondrial dysfunctions characterized by fragmented mitochondrial morphology and apoptotic signaling (186).

The efficacy of VBIT-4 and VBIT-12, VDAC inhibitors, has been documented in treating IBD using DSS- or TNBS-induced colitis animal models (57–59) (**Figure 7**; **Table 3**). Although the detailed mechanism behind VDAC inhibition has been shown to be associated with MAVS and inflammasome activation on intestinal epithelial cells (58), it may normalize mitochondrial calcium imbalance and attenuate effector functions in pro-inflammatory T cells (186).

#### 3.3.3 Mitochondria-associated membrane

Mitochondria and ER are physically connected to form a junction called mitochondria-associated membrane (MAM), which provide a platform for various cellular processes including calcium homeostasis, autophagy, lipid metabolism, and apoptosis. The MAMs contain several tethering molecules such as IP3R, VDAC, and GRP75. In 2018, Bantug and colleagues demonstrated the critical role of MAMs in early activation of effector/memory CD8^+^ T cells (**Figure 7**), which controls effector functions in memory CD8^+^ T cells through an immunometabolic reprogramming that involves mitochondrial respiration, HK binding to VDAC, mTOR/AKT/GSK3b signaling. Inhibition of MAM formation using nocodazole significantly reduces mitochondrial respiration, HK expression, mTOR/AKT/GSK3b signaling pathway, and cytokine secretion (**Figure 7**). However, inhibition of mitochondrial calcium does not affect cytokine production in CD8^+^ T cells (187).

PDK4 has been identified as a novel modulator of MAM integrity and mitochondrial quality control (97). Genetic deletion of PDK4 in activated CD4^+^ T cells significantly diminishes MAM formation, SOCE, and mitochondrial calcium transfer compared to control cells. A novel PDK4 inhibitor, GM-10395, also ameliorates SOCE, mitochondrial calcium, and T cell activation. The anti-inflammatory effects of PDK4 suppression have been demonstrated in DSS-induced colitis animal models and naïve T cell transfer colitis models *in vivo* (49) (**Figure 7**; **Table 3**).

## 4 Future perspectives and conclusion

Over the past few decades, a wealth of scientific knowledge has been generated about the pathophysiology of inflammatory bowel disease (IBD). While the exact mechanisms behind the onset of IBD are complex and subject to debate, immune dysregulation between effector T and regulatory T cells appears to be a major contributing factor.

Experimental studies in IBD have characterized colitogenic T cells, which undergo rapid metabolic reprogramming when exposed to an inflammatory environment. This metabolic adaptation, also known as the *Warburg effect*, was first observed in cancer cells, but recent research has shown that cellular metabolism is closely linked to the activation, differentiation, and functions of CD4^+^ T cells. Additionally, mitochondria play a vital role not only in ATP production, but also in redox balance and Ca^2+^ signaling in T cells.

In this review, we suggest that metabolic regulation, specifically targeting the mitochondrial fitness of CD4^+^ T cells, has the potential to be a next-generation therapy for autoimmune diseases, such as IBD. Supporting this approach, preclinical evidence from animal studies shows that healthy mitochondria from mesenchymal stem cells can redirect the fate of T cells from Th17 effector to Foxp3^+^ Treg cell and can also display immunosuppressive effects on animal models of graft-versus-host disease (188, 189).

Presently, the only clinical trials targeting mitochondria are the MARVEL studies (Mitochondrial Anti-oxidant Therapy to Resolve Inflammation in Ulcerative Colitis), NCT04276740, and NCT05539625, which utilize mitoQ to inhibit mitochondrial ROS. However, other preclinical studies mentioned in this review, which have demonstrated successful results, should also be considered for clinical trials.

Emerging evidence shows that gut microbiota signaling to mitochondria plays a pivotal role in maintaining intestinal homeostasis through metabolic regulation and T-cell activation. Bacterial metabolites, such as short-chain fatty acids and hydrogen sulfide, communicate with colonic epithelial and immune cells, and impact on their metabolic, epigenetic, and genetic functions. However, this topic is not covered here as it is beyond the scope of the present review.

However, autoimmune diseases like IBD are complex and multifactorial, and identifying their etiology can be challenging due to highly variable symptoms and underlying mechanisms. Despite the pathological importance of T cells, other immune cells such as monocytes, macrophages, neutrophils, dendritic cells, and innate lymphoid cells are also implicated in IBD (190). Metabolic reprogramming also plays a significant role in their activation and differentiation (191). Thus, it is important not to overlook the clinical therapeutic approach towards modulating the metabolic regulation of these distinct immune cell populations. In addition, personal genetic backgrounds can also present significant obstacles to developing effective IBD therapeutics (192). Therefore, future studies should focus on developing reliable biomarkers for mitochondrial quality and translating these findings into personalized medication. Moreover, because mitochondrial fitness is believed to be crucial to metabolic homeostasis and energy status, combinatorial strategies using non-drug-based (e.g. diet or lifestyle) or drug-based medications for metabolic syndromes in conjunction with current IBD medications could have a profound impact on patients with IBD, especially those who have reached a plateau in drug efficacy. With continued research in this area, we can look forward to a future where patients with IBD have more effective and personalized treatment options.

## Author contributions

All authors wrote the manuscript. HL wrote the first draft of the manuscript. J-HJ and E-SK wrote sections of the manuscript. All authors contributed to the article and approved the submitted version.
